# Relationships Between Circulating Irisin Response to Ice Swimming and Body Composition in People With Regular Exercise Experience

**DOI:** 10.3389/fphys.2020.596896

**Published:** 2021-01-13

**Authors:** Shuai Mu, Ding Ding, Chao Ji, Qijun Wu, Yang Xia, Long Zhou, Liyu Yang, Gen Ba, Qing Chang, Qin Fu, Yuhong Zhao

**Affiliations:** ^1^Department of Orthopedics, Shengjing Hospital of China Medical University, Shenyang, China; ^2^Department of Clinical Nutrition, Shengjing Hospital of China Medical University, Shenyang, China; ^3^Department of Clinical Epidemiology, Shengjing Hospital of China Medical University, Shenyang, China

**Keywords:** irisin, body composition, fat tissue, skeletal muscle, ice swimming

## Abstract

Severe cold exercise involves the irisin response, and may be related to body composition. We aimed to investigate changes in circulating irisin after ice swimming (IS), as well as to evaluate the correlation between body composition and the change in irisin caused by IS (Δirisin). 81 ice swimmers were recruited to perform IS activities. Blood samples were drawn 30 min before and 30 min after IS, and the serum levels of irisin and the ice swimmers’ body composition were measured. As results, circulating irisin declined significantly during the recovery period following IS exercise (*P* < 0.001). The afternoon baseline circulating irisin level and Δirisin in response to IS were correlated with body fat characteristics rather than muscle parameters in ice swimmers. Δirisin subgroup analyses showed that the Δirisin ascending group (Δirisin+) subjects had a higher fat composition and higher basal irisin levels than the Δirisin descending group (Δirisin−). Furthermore, the decrease in irisin was negatively correlated with fat components in Δirisin− subjects, whereas no correlation was observed between the increase in irisin and body composition in Δirisin + subjects. Finally, a non-linear association analysis suggested that body fat indicators had obvious curvilinear relationships with Δirisin. In conclusion, IS caused a significant decrease in irisin. Statistical and curvilinear associations suggested that the correlation between fat tissue and Δirisin caused by IS is dimorphic and the underlying mechanisms may be due to the different metabolic states of subjects.

## Introduction

Skeletal muscle and adipose tissue are active endocrine organs, which communicate with other organs and affect their metabolism by secreting hormones called myokines and adipokines, respectively, (Polyzos et al., 2018). Irisin is a novel adipo-myokine activated by proteolytic cleavage derived from fibronectin-type III domain-containing 5 (FNDC5) from muscle and adipose tissues in response to exercise (Bostrom et al., 2012; Roca-Rivada et al., 2013). When irisin is secreted into the circulation, it causes the transformation of white adipose tissue into brown adipose tissue by upregulating uncoupling protein 1. This transformation process triggers thermogenesis, increasing energy expenditure and improving metabolism (Bostrom et al., 2012). Hence, irisin has been proposed as an appealing therapeutic target in cases of muscle atrophy and metabolic diseases, such as obesity, diabetes, and non-alcoholic fatty liver disease (Polyzos et al., 2018).

Although it has been established that irisin is produced following physical activity, the effect of exercise on circulating irisin level appears difficult to reconcile (Buscemi et al., 2018; Korta et al., 2019). Numerous studies have shown an increasing effect (Bostrom et al., 2012; Jedrychowski et al., 2015; Zhao et al., 2017; Fox et al., 2018), while others have demonstrated that irisin concentration in the blood does not change or decreases after exercise (Hecksteden et al., 2013; Norheim et al., 2014; Hew-Butler et al., 2015; Qiu et al., 2015). In addition to exercise, cold exposure has been shown to elevate circulatory levels of irisin, which seem to profit from cold-induced muscle shivering thermogenesis (Lee et al., 2014). However, a limited number of studies have evaluated the response of circulating irisin under conditions of simultaneous exercise and cold stimuli.

Irisin is predominantly secreted from skeletal muscle, subcutaneous adipose tissue, and visceral adipose tissue (Roca-Rivada et al., 2013). However, the relationship between circulating irisin and muscle or adipose tissue is still elusive in humans. Some data suggest that circulating irisin level is positively associated with muscle mass, biceps circumference, and muscle strength (Huh et al., 2012; Löffler et al., 2015; Planella-Farrugia et al., 2019). However, other data indicate an inverse association or no correlation between irisin and lean mass (Ellefsen et al., 2014; Winn et al., 2017). Similarly, the majority of studies on adipose tissue support that serum irisin is positively correlated with several parameters of adiposity, such as fat mass (FM), and the waist-to-hip ratio (WHR; Stengel et al., 2013; Löffler et al., 2015). In contrast, other studies have shown that irisin is negatively correlated with these obesity indicators (Ebert et al., 2016).

On the other hand, changes in circulating irisin following exercise training are closely related to body composition. For instance, heavy strength training leads to transient increases in serum irisin concentration, and the response is negatively correlated with the proportion of lean body mass (Nygaard et al., 2015). Nevertheless, whether an association exists in exercise under severe cold stress conditions needs to be evaluated. Ice swimming (IS), which is defined as swimming in water that is 5°C or less (Knechtle et al., 2018), is gaining popularity among people living in high latitude zones and cold regions. IS contains two key factors: exercise and cold exposure. Consequently, IS represents cold exercise.

Accordingly, the aim of this study is to investigate the variations in circulating irisin levels after IS exercise, as well as the relationship between body composition and the change of irisin caused by IS.

## Materials and Methods

### Participants and Study Design

It has been reported that exercise induces increases in muscle FNDC5 expression only in active elderly people, but not in young people (Timmons et al., 2012). Additionally, the majority of ice swimmers are elderly. Hence, the inclusion criteria were IS enthusiasts, not temporary participants, whose ages were >40 years and all volunteered to participate in the study. The exclusion criteria were: (1) Failure to provide informed consent; (2) mental illness and cannot cooperate with the examination; (3) history of cardiovascular or cerebrovascular diseases, such as myocardial infarction, serious arrhythmia, or stroke; (4) taking corticosteroids; and (5) In any other case, participants were considered to have health risks in the study. A total of 90 ice swimmers volunteered to participate in this study. The 90 participants were women and men who were recruited from Liaoyang and Shenyang cities, Liaoning Province, located in Northeast China. Among them, three participants who failed to complete the study and six who had missing irisin results due to a lack of blood samples and detection sensitivity were excluded. Thus, 81 individual results were enrolled in the study.

All participants were divided into three groups, and each group performed the exercise session at a different place and on a different day. The three research times were the winter swimming bases of the Liaoyang ice swimmers on January 12, 2019, and in Shenyang on January 26 and February 16, 2019.

The length of the three study lanes was different, but they were all 25–30 meters. The water temperature during the three winter swimming exercises was <5°C (1, 2, and 1°C, respectively), which earmarked the swimming as IS (Knechtle et al., 2018). On the day of IS, all participants underwent the following in sequence: blood drawn (30 min before IS), performed warm-up preparations onshore, performed IS (14:00 h., according to their respective IS habits; a particular swimming time and distance were not mandatory; however, in this study, exercise intensity was represented by “distance swum per session”), went ashore to perform body temperature recovery exercises, and then had blood drawn (30 min after IS). Warm-up preparation and recovery exercises were performed according to each participant’s habits. These recovery exercises usually included rope skipping, running, and push-ups. Questionnaires were completed and body composition was assessed at the clinical nutrition outpatient department of Shengjing Hospital on the second day of IS. We asked the participants to perform IS according to their own IS distance and time habits on the first day, and reported the distance and time on the questionnaire for the second day. The swimming speed of each participant was different when they were in cold water; however, most participants reported that they took 2–3 min to swim a circle (50–60 meters), and 77.78% of the participants (*n* = 81) swam one circle. Hence, in this study, we did not accurately investigate the swimming time of each participant in icy water; however, the “distance swum per session” obtained from the questionnaires represented to some extent the participants’ swimming time in cold water. This study protocol was approved by the Shengjing Hospital Ethics Committee of China Medical University, and written informed consent was obtained from all participants.

### Questionnaires

Detailed questionnaires were completed by all participants. The questionnaire data included age, name, gender, current smoking behavior, alcohol consumption, diabetes situation, distance swum per session, the frequency of IS per week, and the total years participating in IS.

### Anthropometrics Measurements

The participants were barefoot and wore only underclothes during the body composition measurements. Body composition was assessed using a multi-frequency impedance body composition analyzer (InBody 770; Biospace, Seoul, South Korea). The analyzer provided the following body composition parameters: basal metabolic rate (BMR), body mass index (BMI), FM, percent body fat (FM%), visceral fat area (VFA), WHR, fat free mass (FFM), soft lean mass (SLM), and skeletal muscle mass (SMM). The fat-free mass index (FFMI) was calculated as the FFM divided by height squared (Yang et al., 2019). The skeletal muscle mass index (SMMI) was calculated as the SMM divided by height squared (Yang et al., 2019). The lean-to-fat ratio (LTF) was calculated as the FFM divided by FM (Eisner et al., 2007). The skeletal muscle mass to visceral fat area ratio (SVR) was calculated as the SMM divided by VFA (Hwang et al., 2016). The InBody score is a digital indicator to evaluate body composition based on a scoring system developed by the manufacturer. The score mainly considers body FM and muscle mass, and the system base score is 80 points. If muscle mass exceeds the ideal muscle mass by 1 kg, one point is added to the basic score, and one point is subtracted for each increment less than 1 kg. If the FM exceeds or falls below the ideal FM, 1 kg will be reduced by 1 point. The scoring standards are as follows: A score ≥ 90 indicated that the muscles are well-developed and the body is strong; 70–90 points suggested an ordinary healthy body shape; and ≤70 points indicated weak or obese type, requiring exercise and diet intervention.

### Irisin Measurement

Blood samples were collected by trained nurses between 13:00 h and 15:00 h. Serum samples were obtained by centrifugation and stored at −80°C. Serum irisin levels were determined by commercial enzyme-linked immunosorbent assay (ELISA) kits following the manufacturer’s instructions (CSB-EQ027943HU, Cusabio Biotech, Wuhan, China). The sensitivity of the irisin assay was 0.78 ng/mL, and the intra-assay coefficient of variation was <8%. The “irisin before” samples represented the circulating irisin levels before IS (baseline irisin level). The “irisin after” samples represented the circulating irisin levels after IS. The Δirisin represented the change in circulating irisin level in response to IS. Δirisin + represented the Δirisin ascending group (Δirisin > 0) and Δirisin− represented the Δirisin descending group (Δirisin < 0).

### Statistical Analysis

**FIGURE 1:**
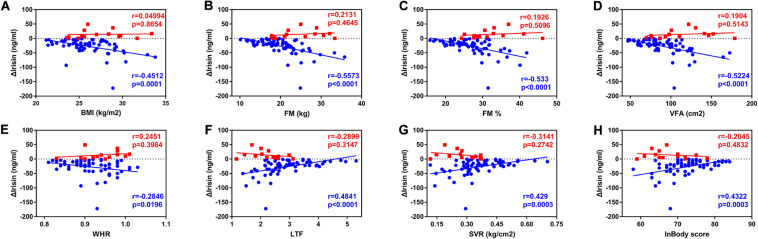
Correlations between anthropometric characteristics and the Δirisin+ and the Δirisin− groups. **(A)** Negative correlation between body mass index (BMI) and Δirisin−; **(B)** negative correlation between fat mass (FM) and Δirisin−; **(C)** negative correlation between percent body fat (FM%) and Δirisin−; **(D)** negative correlation between visceral fat area (VFA) and Δirisin−; **(E)** negative correlation between the waist to-hip ratio (WHR) and Δirisin−; **(F)** positive correlation between the lean-to-fat ratio (LTF) and Δirisin−; **(G)** positive correlation between skeletal muscle mass and the visceral fat area ratio (SVR) and Δirisin−; and **(H)** positive correlation between the InBody score and Δirisin−.

Data of continuous variables are presented as mean and standard deviation. Data of categorical variables are presented as numbers and percentages. The paired *t*-test was used to detect differences in serum irisin levels before and after IS. Multiple linear regression analysis models were used to test the relationship between the anthropometric characteristics and baseline irisin and Δirisin levels after adjusting for age and gender in model I, and after adjusting for age, gender, smoking, alcohol drinking, diabetes, distance swum per session, the frequency of IS per week, and the total years participating in IS in Model II. Student’s *t*-test was used to detect differences in age and anthropometric characteristics between the Δirisin+ and Δirisin− groups. Associations between BMI, FM, FM%, VFA, WHR, LTF, SVR, and the InBody score with Δirisin+ and Δirisin− levels were determined by Pearson’s correlation coefficient analysis. Smooth curve fitting was conducted to determine the non-linear relationships between fat body composition variables and Δirisin level after adjusting for age, gender, smoking, alcohol drinking, diabetes, distance swum per session, the frequency of IS per week, and the total years participating in IS. A two-sided *P*-value < 0.05 was considered significant. All analyses were performed using EmpowerStats software^1^ and the statistical package R (3.4.3) version (The R Foundation for Statistical Computing, Vienna, Austria). Figure 1 data were depicted by GraphPad Prism software (GraphPad Inc., La Jolla, CA, United States).

## Results

### Demographical and Clinical Characteristics of the Study Population

As shown in Table 1, the mean age of the participants was 58.3 ± 8.4 years, and their average BMI was 26.2 ± 2.7 kg/m^2^. According to the criteria of the World Health Organization (2000), no underweight (BMI < 18.5 kg/m^2^) volunteers participated in the study. The participants’ body composition parameters mainly included fat-related parameters and muscle-related parameters. The baseline irisin level was 104.4 ± 100.9 ng/ml, and the irisin level after IS was 84.7 ± 94.6 ng/ml. The change in irisin (Δirisin) following IS was −19.7 ± 29.7 ng/ml. Furthermore, a significant difference was found in the change in irisin after IS (*P* < 0.001). The mean value of the Δirisin + group was 14.3 ± 14.4 ng/ml, while that of the Δirisin− group was −26.9 ± 27.1 ng/ml.

**TABLE 1:** Baseline characteristics of participants (*N* = 81).

**Characteristic**	**Statistics**	**Range**
Men, *n* (%)	66 (81.5)	
Age, (years)	58.3 ± 8.4	42.0–84.0
Current smoker, *n* (%)	20 (24.7)	
Alcohol consumption (≥30 g/day), *n* (%)	42 (51.9)	
Diabetes, *n* (%)	7 (8.6)	
Ice swimming distance per session, *n* (%), (meters)		30–250
<80 m	69 (85.2)	
≥80 m	12 (14.8)	
Ice swimming frequency per week, *n* (%)		1.0–7.0
≤3 t/w	14 (17.3)	
4–5 t/w	25 (30.9)	
6–7 t/w	42 (51.9)	
Total Ice swimming years, *n* (%), (years)		2–25
<5 years	30 (37.0)	
5–9 years	24 (29.6)	
≥10 years	27 (33.3)	
BMR, (kcal)	1536.8 ± 177.7	1074.0–1828.0
BMI, (kg/m^2^)	26.2 ± 2.7	21.2–33.8
FM, (kg)	20.7 ± 5.4	10.1–35.8
FM%	27.6 ± 5.9	15.8–46.2
VFA, (cm^2^)	93.4 ± 28.4	47.2–178.9
WHR	0.9 ± 0.1	0.8–1.0
FFM, (kg)	54.0 ± 8.2	32.6–67.5
FFMI, (kg/m^2^)	18.9 ± 1.8	14.1–22.5
SLM, (kg)	51.0 ± 7.8	30.7–63.7
SMM, (kg)	29.9 ± 5.5	8.3–38.4
SMMI, (kg/m^2^)	10.4 ± 1.4	3.0–12.9
LTF	2.8 ± 0.8	1.2–5.3
SVR, (kg/cm^2^)	0.3 ± 0.1	0.1–0.7
Inbody score	71.7 ± 5.7	58.0–84.0
Irisin before, (ng/ml)	104.4 ± 100.9	9.2–632.0
Irisin after, (ng/ml)	84.7 ± 94.6	1.8–647.3
Δirisin, (ng/ml)	−19.7 ± 29.7	−172.0–49.0
Δirisin+, (ng/ml)	14.3 ± 14.4	0.5–49.0
Δ irisin−, (ng/ml)	−26.9 ± 27.1	−172.0–−0.7

*Continuous variables are represented by Mean ± standard deviation; Categorical variables are represented by numerical and percentage.*

*Abbreviations: BMR, basal metabolic rate; BMI, body mass index; FM, fat mass; FM%, percent body fat; VFA, visceral fat area; WHR, waist to hip ratio; FFM, fat free mass; FFMI, fat free mass index; SLM, soft lean mass; SMM, skeletal muscle mass; SMMI, skeletal muscle mass index; LTF, lean-to-fat ratio; and SVR, skeletal muscle mass to visceral fat area ratio. Irisin before, the circulating irisin levels before ice swimming (baseline irisin levels); Irisin after, the circulating irisin levels after ice swimming; Δirisin, the circulating irisin changes responses to ice swimming; Δirisin+, the Δirisin ascending group; and Δ irisin−, the Δirisin descending group.*

### Correlations Between the Anthropometric Characteristics and Baseline Irisin Level

In multiple regression analyses, the unadjusted results revealed a positive correlation between BMI, FM, FM%, VFA, and WHR and the baseline irisin level (Table 2). A negative correlation was observed between FFM, SLM, LTF, SVR, the InBody score, and baseline irisin level. However, the association between FFM, SLM, and baseline irisin level vanished after adjusting for covariates (Table 2). Furthermore, no correlation was detected between BMR, FFMI, SMM, and SMMI and the baseline irisin level (Table 2). It should be noted here that the WHR data obtained by bioimpedance are less accurate than those collected with classical methods (Abedi Yekta et al., 2016).

**TABLE 2:** Multivariate linear regression analysis of the associations of anthropometrical characteristics and irisin baseline levels.

**Variable**	**Crude model**		**Model I**		**Model II**	
	**β (95% CI)**	***P*-value**	**β (95% CI)**	***P*-value**	**β (95%CI)**	***P*-value**
BMR	−0.15 (−0.27, −0.03)	0.0186*	0.06 (−0.14, 0.25)	0.5544	0.05 (−0.16, 0.27)	0.6262
BMI	18.35 (11.23, 25.46)	< 0.0001***	21.98 (15.76, 28.19)	< 0.0001***	23.51 (16.82, 30.21)	< 0.0001***
FM	12.60 (9.54, 15.65)	< 0.0001***	11.99 (9.17, 14.81)	< 0.0001***	12.91 (9.93, 15.88)	< 0.0001***
FM%	12.72 (10.20, 15.23)	< 0.0001***	13.10 (10.05, 16.14)	< 0.0001***	14.16 (10.98, 17.34)	< 0.0001***
VFA	2.55 (2.01, 3.10)	< 0.0001***	2.38 (1.86, 2.90)	< 0.0001***	2.53 (1.99, 3.08)	< 0.0001***
WHR	696.07 (302.96, 1089.19)	0.0008***	1024.52 (678.78, 1370.26)	< 0.0001***	1066.10 (697.19, 1435.02)	< 0.0001***
FFM	−3.20 (−5.81, −0.59)	0.0186*	1.27 (−2.94, 5.49)	0.5560	1.16 (−3.52, 5.85)	0.6276
FFMI	−7.70 (−20.24, 4.83)	0.2319	13.56 (−2.95, 30.08)	0.1115	13.10 (−5.00, 31.19)	0.1605
SLM	−3.43 (−6.18, −0.68)	0.0167*	1.28 (−3.21, 5.76)	0.5789	1.14 (−3.84, 6.13)	0.6547
SMM	−3.61 (−7.54, 0.32)	0.0758	2.13 (−3.03, 7.29)	0.4213	2.04 (−3.58, 7.66)	0.4796
SMMI	−5.05 (−20.81, 10.71)	0.5320	12.64 (−4.61, 29.89)	0.1550	12.25 (−6.41, 30.91)	0.2024
LTF	−78.85 (−99.49, −58.22)	< 0.0001***	−73.18 (−96.66, −49.70)	< 0.0001***	−81.26 (−106.63, −55.89)	< 0.0001***
SVR	−536.76 (−688.78, −384.73)	< 0.0001***	−489.44 (−659.80, −319.08)	< 0.0001***	−521.75 (−704.36, −339.15)	< 0.0001***
InBody score	−9.44 (−12.76, −6.13)	< 0.0001***	−9.64 (−12.82, −6.46)	< 0.0001***	−10.51 (−13.87, −7.15)	< 0.0001***

Abbreviations: Crude, no adjustment; Model I, adjusted for age and gender; Model II, adjusted for age, gender age, gender, smoke, alcohol drinking, diabetes, distance swum per session, the frequency of ice swimming every week, and the total years of ice swimming; β, regression coefficient; CI, confidence interval; BMR, basal metabolic rate; BMI, body mass index; FM, fat mass; FM%, percent body fat; VFA, visceral fat area; WHR, waist to hip ratio; FFM, fat free mass; FFMI, fat free mass index; SLM, soft lean mass; SMM, skeletal muscle mass; SMMI, skeletal muscle mass index; LTF, lean-to-fat ratio; and SVR, skeletal muscle mass to visceral fat area ratio. *P < 0.05 and ***P < 0.001.

### Correlations Between the Anthropometric Characteristics and Δirisin Level

As shown in Table 3, BMI was negatively correlated with Δirisin after adjusting for age and gender. The FM% was negatively correlated with Δirisin, and LTF was positively correlated with Δirisin after adjusting for age, gender, smoking, alcohol drinking, diabetes, distance swum per session, frequency of IS every week, and total years participating in IS.

**TABLE 3:** Multivariate linear regression analysis of the associations of anthropometrical characteristics and Δirisin levels.

**Variable**	**Crude model**		**Model I**		**Model II**	
	**β (95%CI)**	***P*-value**	**β (95%CI)**	***P*-value**	**β (95%CI)**	***P*-value**
BMR	−0.01 (−0.04, 0.03)	0.7441	−0.01 (−0.07, 0.05)	0.7113	−0.00 (−0.07, 0.07)	0.9095
BMI	−2.34 (−4.69, 0.02)	0.0555	−2.60 (−5.06, -0.15)	0.0412*	−2.75 (−5.47, −0.03)	0.0515
FM	−1.15 (−2.34, 0.04)	0.0627	−1.21 (−2.42, 0.00)	0.0539	−1.30 (−2.63, 0.03)	0.0602
FM%	−0.85 (−1.94, 0.24)	0.1319	−1.33 (−2.64, −0.01)	0.0514	−1.49 (−2.93, −0.05)	0.0462*
VFA	−0.18 (−0.40, 0.05)	0.1314	−0.19 (−0.43, 0.04)	0.1126	−0.20 (−0.45, 0.06)	0.1378
WHR	−44.80 (−168.75, 79.15)	0.4808	−43.80 (−175.62, 88.02)	0.5169	−38.75 (−181.91, 104.41)	0.5974
FFM	−0.13 (−0.93, 0.66)	0.7430	−0.26 (−1.60, 1.09)	0.7101	−0.09 (−1.59, 1.42)	0.9091
FFMI	−1.58 (−5.29, 2.13)	0.4058	−3.06 (−8.37, 2.24)	0.2615	−2.85 (−8.71, 3.01)	0.3440
SLM	−0.14 (−0.98, 0.70)	0.7465	−0.27 (−1.70, 1.16)	0.7154	−0.08 (−1.69, 1.52)	0.9193
SMM	−0.18 (−1.36, 1.00)	0.7693	−0.22 (−1.87, 1.43)	0.7949	0.09 (−1.72, 1.90)	0.9258
SMMI	−1.45 (−6.09, 3.20)	0.5429	−1.86 (−7.41, 3.70)	0.5147	−1.02 (−7.08, 5.04)	0.7428
LTF	6.42 (−1.40, 14.24)	0.1118	8.75 (−0.16, 17.66)	0.0580	10.27 (0.34, 20.19)	0.0464*
SVR	39.35 (−16.75, 95.45)	0.1731	52.25 (−11.22, 115.72)	0.1107	61.48 (−7.77, 130.73)	0.0863
Inbody score	0.64 (−0.51, 1.78)	0.2792	0.62 (−0.59, 1.84)	0.3192	0.75 (−0.58, 2.08)	0.2739

Abbreviations: Δirisin, the circulating irisin changes responses to ice swimming; Crude, no adjustment; Model I, adjusted for age and gender; Model II, adjusted for age, gender age, gender, smoke, alcohol drinking, diabetes, distance swum per session, the frequency of ice swimming every week, and the total years of ice swimming; β, regression coefficient; CI, confidence interval; BMR, basal metabolic rate; BMI, body mass index; FM, fat mass; FM%, percent body fat; VFA, visceral fat area; WHR, waist to hip ratio; FFM, fat free mass; FFMI, fat free mass index; SLM, soft lean mass; SMM, skeletal muscle mass; SMMI, skeletal muscle mass index; LTF, lean-to-fat ratio; and SVR, skeletal muscle mass to visceral fat area ratio. *P < 0.05.

### Differences in the Anthropometric Characteristics of the Δirisin+ and the Δirisin− Groups

The study subjects were divided into the Δirisin ascending group (Δirisin+) and the Δirisin descending group (Δirisin−). We compared the differences in age, anthropometric characteristics, and the baseline irisin level between the two groups (Table 4). The Δirisin + group of subjects had significantly higher FM, FM%, VFA, and baseline irisin level than those of the Δirisin− control subjects. In contrast, LTF, SVR, and the InBody score of the Δirisin + group subjects were lower than those of the Δirisin− control subjects. Notably, no underweight (BMI < 18.5 kg/m^2^) volunteers participated; “InBody score” of 58–84 (Table 1). Hence, according to the scoring standard, a lower InBody score represented more fat and less muscle in this study (data not shown).

**TABLE 4:** The differences in the anthropometrical characteristics of the Δirisin+ and the Δirisin− groups.

**Characteristic**	**Δirisin− (*N* = 67)**	**Δirisin+ (*N* = 14)**	***P*-value**
Age, (years)	58.36 ± 8.05	57.79 ± 10.19	0.818
BMR, (kcal)	1547.57 ± 173.14	1485.21 ± 196.83	0.235
BMI, (kg/m^2^)	25.93 ± 2.62	27.40 ± 2.97	0.066
FM, (kg)	19.90 ± 5.05	24.40 ± 5.56	0.004**
FM%	26.70 ± 5.45	32.14 ± 6.14	0.001**
VFA, (cm^2^)	89.25 ± 25.67	113.09 ± 33.17	0.004**
WHR	0.91 ± 0.05	0.94 ± 0.05	0.052
FFM, (kg)	54.52 ± 8.02	51.62 ± 9.11	0.233
FFMI, (kg/m^2^)	18.94 ± 1.76	18.49 ± 1.79	0.388
SLM, (kg)	51.51 ± 7.61	48.76 ± 8.60	0.233
SMM, (kg)	30.10 ± 5.56	28.76 ± 5.54	0.415
SMMI, (kg/m^2^)	10.44 ± 1.46	10.29 ± 1.18	0.719
LTF	2.910.82	2.210.56	0.003**
SVR, (kg/cm^2^)	0.36 ± 0.12	0.27 ± 0.08	0.009**
Inbody score	72.51 ± 5.40	67.64 ± 5.42	0.003**
Irisin before, (ng/ml)	93.66 ± 80.00	155.94 ± 163.39	0.035*

Continuous variables are represented by Mean ± standard deviation. Abbreviations: Δirisin+, the Δirisin ascending group; Δ irisin−, the Δirisin descending group; BMR, basal metabolic rate; BMI, body mass index; FM, fat mass; FM%, percent body fat; VFA, visceral fat area; WHR, waist to hip ratio; FFM, fat free mass; FFMI, fat free mass index; SLM, soft lean mass; SMM, skeletal muscle mass; SMMI, skeletal muscle mass index; LTF, lean-to-fat ratio; and SVR, skeletal muscle mass to visceral fat area ratio; Irisin before, the circulating irisin levels before ice swimming (baseline irisin levels). *P < 0.05 and **P < 0.01.

### Relationships Between Anthropometric Characteristics and the Δirisin+ and the Δirisin− Groups

Spearman’s correlation analyses demonstrated that BMI, FM, FM%, VFA, and WHR were negatively associated with Δirisin− (rBMI = −0.4512, *P* = 0.0001; rFM = −0.5573, *P* < 0.0001; rFM% = −0.533, *P* < 0.0001; rVFA = −0.5224, *P* < 0.0001; rWHR = −0.2846, and *P* = 0.0196). In contrast, the LTF, SVR, and the InBody score were positively associated with Δirisin− (rLTF = 0.4841, *P* < 0.0001; rSVR = 0.429, *P* = 0.0003; rInBody score = 0.4322, and *P* = 0.0003). No correlations were found between the anthropometric characteristics and Δirisin+ (Figure 1).

### Non-linear Relationship Between Anthropometric Characteristics and Δirisin Levels

**FIGURE 2:**
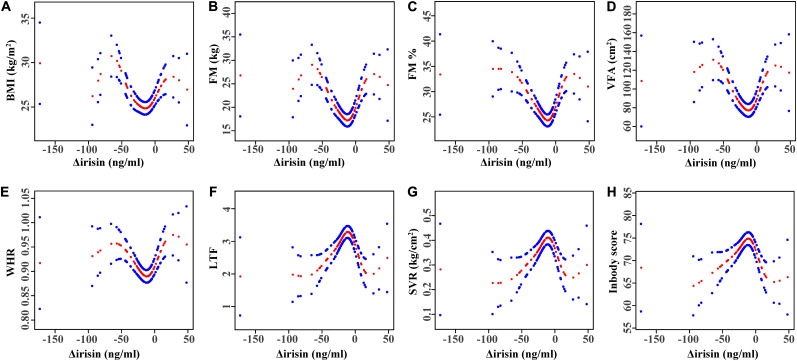
Multivariate adjusted smoothing spline plots of anthropometric characteristics by Δirisin level. **(A)** Relationship between Δirisin levels and body mass index (BMI); **(B)** Δirisin levels with fat mass (FM); **(C)** Δirisin levels with percent body fat (FM%); **(D)** Δirisin levels with visceral fat area (VFA); **(E)** Δirisin levels with the waist-to-hip ratio (WHR); **(F)** Δirisin levels with the lean-to-fat ratio (LTF); **(G)** Δirisin levels with the skeletal muscle mass to visceral fat area ratio (SVR); and **(H)** Δirisin levels with the InBody score; The adjusted factors were age, gender, smoking, alcohol drinking, diabetes, distance swum per session, frequency of IS per week, and total years participating in IS. Red dotted lines represent the spline plots of Δirisin levels and blue dotted lines represent the 95% confidence intervals of the spline plots.

Smooth curve fitting was performed after adjusting for covariates. Figure 2 shows the *U*-shaped relationship between BMI, FM, FM%, VFA, WHR, and Δirisin level. The threshold analysis for the relationship between BMI, FM, FM%, VFA, WHR, and Δirisin level, and the turning points in a two-piecewise regression model in the Δirisin level was shown in Table 5. A negative relationship trend was found between BMI, FM, FM%, VFA, and WHR and Δirisin levels when the Δirisin level was lower than the turning point value; but a positive association was detected between BMI, FM, FM%, VFA, and WHR and Δirisin level when Δirisin was beyond the turning point value. In contrast, there was an inverted *U*-curve relationship between LTF, SVR, and the InBody score and the Δirisin level (Figure 2). The turning point in the Δirisin level was −8.3 ng/ml (Table 5).

**TABLE 5:** Piece-wise linear regression analysis for the relationship between anthropometrical characteristics and Δirisin levels.

**Variables**	**Δirisin adjusted^#^**	
	**β (95%CI)**	***P-*value**
**BMI**		
Δirisin < −10.93 ng/ml	−0.05 (−0.07, −0.02)	0.0003***
Δirisin > −10.93 ng/ml	0.07 (0.01, 0.12)	0.0175*
**FM**		
Δirisin < −8.3 ng/ml	−0.10 (−0.15, −0.06)	< 0.0001***
Δirisin > −8.3 ng/ml	0.21 (0.10, 0.32)	0.0003***
**FM%**		
Δirisin < −8.46 ng/ml	−0.10 (−0.14, −0.06)	< 0.0001***
Δirisin > −8.46 ng/ml	0.21 (0.11, 0.30)	0.0001***
**VFA**		
Δirisin < −8.3 ng/ml	−0.47 (−0.71, −0.23)	0.0002***
Δirisin > −8.3 ng/ml	1.06 (0.46, 1.66)	0.0009***
**WHR**		
Δirisin < −8.46 ng/ml	−0.00 (−0.00, −0.00)	0.0168*
Δirisin > −8.46 ng/ml	0.00 (0.00, 0.00)	0.0045**
**LTF**		
Δirisin < −8.3 ng/ml	0.01 (0.01, 0.02)	< 0.0001***
Δirisin > −8.3 ng/ml	−0.03 (−0.04, −0.01)	0.0006***
**SVR**		
Δirisin < −8.3 ng/ml	0.00 (0.00, 0.00)	0.0003***
Δirisin > −8.3 ng/ml	−0.00 (−0.01, −0.00)	0.0041**
**InBody score**		
Δirisin < −8.3 ng/ml	0.09 (0.04, 0.13)	0.0004***
Δirisin > −8.3 ng/ml	−0.23 (−0.34, −0.11)	0.0002***

*Δirisin, the circulating irisin changes responses to ice swimming; #, indicates adjusted for age, gender age, gender, smoke, alcohol drinking, diabetes, distance swum per session, the frequency of ice swimming every week, and the total years of ice swimming.*

Abbreviations: β, regression coefficient; CI, confidence interval; BMI, body mass index; FM, fat mass; FM%, percent body fat; VFA, visceral fat area; WHR, waist to hip ratio; LTF, lean-to-fat ratio; and SVR, skeletal muscle mass to visceral fat area ratio.

*P < 0.05; **P < 0.01; and ***P < 0.001.

## Discussion

Since its discovery, irisin has received substantial attention from the scientific community, as it may benefit metabolic disorders. Interestingly, exercise and cold exposure are key triggers activating irisin release (Bostrom et al., 2012; Lee et al., 2014). However, to date, the role of severe cold exercise in the association between variations in irisin and body composition in humans has remained elusive. In the present study, we chose IS as a typical severe cold exercise to explore these questions. We recruited 81 ice swimmers, which is the largest IS study to have been performed with usual IS activities. As results, circulating irisin levels unexpectedly decreased significantly after IS. The afternoon baseline circulating irisin level was positively correlated with body fat characteristics, whereas it was inversely correlated with the skeletal muscle parameters and the ratio of muscle to fat in ice swimmers. FM% and LTF were significantly correlated with Δirisin level after adjusting for covariates. However, when we further analyzed the differences between the groups of increasing and decreasing Δirisin, the Δirisin + group subjects had higher fat composition and a higher basal irisin level than those of the Δirisin− group subjects. Furthermore, among the Δirisin− group subjects, some body fat variables were negatively associated with Δ irisin−, whereas no correlation was observed between the change in irisin and body composition in the Δirisin + group subjects. Finally, we applied curvilinear correlation to determine the relationship between body fat composition and Δirisin. We show, for the first time, that some body fat indicators had curvilinear relationships with Δirisin.

In contrast to our hypothesis that exercise in a cold environment elicits a marked increase in circulating irisin levels, the results of this study show that the circulating irisin levels of ice swimmers were significantly lower after the IS cold stress. These results are consistent with the findings of Bubak et al. (2017), who reported that exercise in a cold environment does not enhance circulating irisin levels and that there is a significant decrease in irisin level from just after exercise to 3 h post-exercise when not considering plasma volume shifts regardless of temperature. There is currently no plausible interpretation for this paradox. However, the changes in irisin may be related to the source and metabolism of irisin; hence, they may be related to body composition. In this study, we investigated the relationship between Δirisin, fat, muscle, and the ratio of muscle to fat. As results, FM% was inversely correlated with Δirisin, while LTF was positively correlated with Δirisin, suggesting that different body compositions, particularly obesity status, can affect the variations in irisin levels caused by IS. In addition, other potential reasons for irisin decline after IS, such as metabolic rates and metabolic milieu (glucose, insulin, and fatty acid) changes were not confirmed as there were large and fairly consistent individual differences in cold acclimatization (Keatinge, 1960), however, future studies are needed to substantiate the intriguing possibility.

The second finding of this study is that the fat components of the body, but not the muscle components, were positively correlated with the basal circulating irisin level. This conclusion is consistent with literature reports, which support that serum irisin is positively correlated with fat tissue (Stengel et al., 2013; Löffler et al., 2015; Polyzos et al., 2018). Specifically, in this study, ice swimmers without lean constitution and preliminary differences between participants may be part of the reason behind the associations. The underlying mechanisms of the positive correlation between irisin and fat tissue may be the same as those speculated in previous reports: (1) Although irisin in the blood may be mainly secreted by muscle, with the increase in fat, the amount of secreted irisin from adipose tissue is probably higher than that in a lean state due to the increase in total FM (Perakakis et al., 2017; Polyzos et al., 2018); (2) the higher irisin levels detected in obese individuals might represent a compensatory mechanism to increase energy expenditure and improve insulin sensitivity and glucose homeostasis to achieve metabolic balance, which can also be called “irisin resistance” (Polyzos et al., 2013; Roca-Rivada et al., 2013; Perakakis et al., 2017). In addition, it is worth noting that the baseline irisin level in our study was measured in the afternoon without fasting. The reason for this is that most ice swimmers IS in the afternoon. So irisin measured at this time is a more realistic baseline level before IS. Few studies have investigated irisin during the afternoon (Winn et al., 2017), and the impact of diet and circadian rhythms on irisin levels remains controversial (Anastasilakis et al., 2014; Löffler et al., 2015). Nevertheless, our study has shown that the relationship between fat composition and irisin remained in the afternoon.

The more intriguing finding of this study was obtained by dividing Δirisin into the Δirisin+ and Δirisin− subgroups. The results show that the Δirisin + group subjects had significantly higher FM, FM%, VFA, WHR, and baseline circulating irisin levels, but lower LTF, SVR, and InBody score than those of the Δirisin− control subjects (Table 4), suggesting that different body composition states may cause different changes in circulating irisin after IS. In the few studies that have investigated the influence of cold on irisin concentration, Dulian et al. (2015) reported that whole-body cryostimulation increases blood irisin levels in non-active obese men, whereas it causes a decrease in irisin in active obese men. In Dulian’s study, the non-active obese men represented subjects with a low fitness level, which reflects higher fat composition (FM, FM%, and VFA) and lower muscle (FFM%) than those in obese active men (Dulian et al., 2015). These results are consistent with our Δirisin + group and Δirisin− group results. The Δirisin + group of subjects may represent a low fitness level, whereas the Δirisin− group was composed of high fitness level subjects, and the two different populations showed different changes in circulating irisin when exposed to cold. This phenomenon is also consistent with a previous hypothesis, in which individual differences in responsiveness to the action of irisin after exercise may reflect the energy/ATP status of the muscle (Polyzos et al., 2013). In this regard, irisin adjusts energy expenditure and metabolism preferentially in individuals who are less trained, whereas the increase in irisin levels with exercise may be less significant among subjects who are well trained and/or who exercise regularly (Huh et al., 2012). These data suggest that relatively “well trained people” do not produce more, or may produce less circulating irisin during cold exposure or exercise to achieve an energy balance. Accordingly, most of the participants in this study were long-term ice swimmers, who trained regularly. As a result, we observed a significant decrease in irisin after IS.

Among the Δirisin− group subjects, the decrease in irisin was negatively correlated with fat components, suggesting that adipose tissue may be related to the decrease in serum irisin after IS. Irisin is excreted by through the liver and kidneys (Lv et al., 2015). However, the potential mechanism for eliminating irisin as it relates to fat tissue needs to be elucidated. It has been reported that irisin can be taken up by some cells, particularly adipocytes (Tine Kartinah et al., 2018). In contrast, it is also possible that fat tissue may indirectly affect the decrease in irisin. Here, we speculate that there was an unidentified factor released by adipocytes in the Δirisin− group subjects (relatively “well trained people”), which may limit irisin or cause a compensatory reduction in circulating irisin. As a result, the more fat in the Δirisin− subjects, the greater the decrease in circulating irisin level. For instance, the adipokine adiponectin is induced by exercise or cold exposure (Imbeault et al., 2009; Yu et al., 2017), and is negatively correlated with irisin level (Nigro et al., 2017). However, this hypothesis needs to be confirmed.

Lastly, we explored whether there were non-linear correlations between anthropometric characteristics and Δirisin levels. Interestingly, a curvilinear correlation was detected between the fat composition variables and Δirisin level. A *U*-shaped relationship was found between BMI, FM, FM%, VFA, and WHR and Δirisin level, whereas there was an inverted *U*-curve relationship between LTF, SVR, and the InBody score and Δirisin level (Figure 2). These curved relationships demonstrate the difference between Δirisin levels and body composition in the Δirisin− and Δirisin + groups. The curvilinear relationship seems to indicate the true relationship between body composition and Δirisin, and suggests that subjects with high body fat may increase circulating irisin level after IS, and may also cause a significant decrease in irisin, which could be related to individual differences. In addition, the bidirectional curve may also suggest why there is considerable controversy in the study of irisin. For instance, contradictory evidence has been reported associating irisin circulating levels with physical activity, body composition, glucose, insulin resistance, and metabolic syndrome (Perakakis et al., 2017; Polyzos et al., 2018). Furthermore, Kim et al. reported that irisin may have dual effects on anabolism and catabolism to the skeleton, similar to parathyroid hormone (Kim et al., 2018), which suggests the bidirectionality of irisin. Accordingly, further studies that emphasize the threshold and/or saturation effects of irisin are needed.

This study had several limitations. First, we used commercial ELISA kits to detect circulating irisin levels. Although ELISA kits are one of the five most widely applied kits to study irisin (Perakakis et al., 2017), ELISA kits may lack assay specificity. Second, the gold standard tool to assess body composition is dual-energy *X*-ray absorptiometry. However, the Inbody measurement system is also a precise and convenient way to measure body composition (Hwang et al., 2016). Third, we only investigated serum irisin once in 30 min after IS, so the results do not represent the overall irisin response during the recovery period after IS. However, the reason for this design was to reduce the effect of the irisin circadian rhythm on the results (Anastasilakis et al., 2014), and it has reported that 30 min is the sufficient time for stimulation irisin secretion by exercise or cryostimulation intervention (Huh et al., 2012; Dulian et al., 2015; Szumilewicz et al., 2019). Lastly, no data on metabolic disorders, the time interval between lunch and IS, diet on the test day, cardiorespiratory fitness measurements, or detection of *in vivo* metabolic parameters were included in our study, so we could not adjust for metabolic pathologies or feeding effects that may associated with energy balance and the evaluation of macronutrients status to distinguish the exact metabolic states of the Δirisin− and Δirisin + groups.

In conclusion, the present study was conducted in a group of ice swimmers and showed that circulating irisin declined after IS. Concurrently, the current data indicate that the baseline irisin level in the afternoon and the change in circulating irisin in response to IS are correlated with the body fat characteristics of ice swimmers. The correlation between fat tissue and Δirisin caused by IS is dimorphic and the underlying mechanisms may be due to the different metabolic states of subjects, resulting in different irisin responses to achieve energy balance. However, further studies are needed to clarify these results.

## Data Availability Statement

The raw data supporting the conclusions of this article will be made available by the authors, without undue reservation.

## Ethics Statement

The studies involving human participants were reviewed and approved by Shengjing Hospital Ethics Committee of China Medical University. The patients/participants provided their written informed consent to participate in this study.

## Author Contributions

SM, YX, and YZ designed and supervised the study. SM, DD, and CJ enrolled participants, performed exercise testing, and supervised the training interventions. SM, CJ, YX, QW, and QC analyzed training and testing data, interpreted results, and wrote the manuscript. LZ, LY, GB, and QF reviewed the manuscript and provided important intellectual content. All authors approved the final version of the manuscript.

## Conflict of Interest

The authors declare that the research was conducted in the absence of any commercial or financial relationships that could be construed as a potential conflict of interest.

## Footnotes

1 http://www.empowerstats.com/en/features.html
